# Volunteering, income and health

**DOI:** 10.1371/journal.pone.0173139

**Published:** 2017-03-08

**Authors:** Jens Detollenaere, Sara Willems, Stijn Baert

**Affiliations:** 1 Department of Family Medicine and Primary Health Care, Ghent University, Ghent, Belgium; 2 Department of Social Economics, Ghent University, Ghent, Belgium; 3 Research Foundation – Flanders, Brussels, Belgium; 4 Department of Sociology, University of Antwerp, Antwerp, Belgium; 5 IRES, Université catholique de Louvain, Louvain-la-neuve, Belgium; 6 Institute for the Study of Labor (IZA), Bonn, Germany; Middlesex University, UNITED KINGDOM

## Abstract

Separate literatures have related volunteering to health gains and income gains. We study the association between volunteering, income and health within one statistical framework. A state-of-the-art mediation analysis is conducted on data concerning the health, volunteering and sociodemographic characteristics of 42926 individuals within 29 European countries. We find that volunteering is positively associated to self-rated health. This association is partially mediated by household income.

## 1. Introduction

Volunteering is a widespread activity in the adult population of many OECD countries [1–2]. More than 23% of the respondents in the sixth round of the European Social Survey (ESS6), gathered in 2012 and 2013, reported to be involved in work for voluntary organisations at least once every six months. Although volunteering activities are inherently targeted at creating benefits for others, over the past decades, there has been considerable debate in peer-reviewed literature about whether these activities are also associated with benefits for the volunteers themselves.

Scholars in health and (other) social sciences have linked volunteer work to beneficial health-related outcomes (for instance, with respect to self-rated health, functional limitations, health behaviours, depression and mortality). From a theoretical point of view, volunteering may improve access to psychological resources (such as self-esteem and self-efficacy) and social resources (social integration and, *ipso facto*, access to support and information), both of which are found to have an overall positive effect on health [3–5]. In addition, volunteering increases physical and cognitive activity, which protects against functional decline and dementia in old age [6]. Finally, neuroscience research has related volunteering to the release of the caregiving-related hormones oxytocin and progesterone, which have the capacity to regulate stress and inflammation [7]. From an empirical point of view, evidence for a positive association between volunteering and health has been found in different age groups in countries such as Canada, Germany, Israel, Spain, Taiwan, the United Kingdom and the United States [1, 3–6, 8–19].

In parallel, economists have investigated the economic surplus of volunteering. More concretely, Day and Devlin [20], Day and Devlin [21], Prouteau and Wolff [22], Hackl et al. [23], Cozzi et al. [24] and Sauer [25] found statistically significantly positive effects of volunteer work on income based on administrative and survey data for Austria, Canada, France, the United Kingdom and the United States. Given this evidence and the well-established positive association between income and health [19, 26–31], one could expect that volunteering has, besides its aforementioned direct effect, also an indirect beneficial effect on health outcomes via income.

In the present study, we empirically investigate within one statistical framework the direct association between volunteering and self-rated health and their indirect association via income. We use data about the volunteering, health and sociodemographic characteristics of 42926 individuals within 29 countries covered by ESS6. These data are analysed by means of a regression analysis controlling for country fixed effects and a mediation analysis. By measuring the aforementioned direct and indirect associations, keeping sociodemographic background constant, we test the following hypotheses, which are supported by the reviewed literatures:

**H1:** Volunteering is directly associated with higher self-rated health.

**H2:** Volunteering is indirectly associated with higher self-rated health via household income.

The remainder of this study is structured as follows. In the following section, we present our data and provide the reader with a descriptive analysis of these data. In addition, in this section our statistical models are introduced. Section 3 reports our main estimation results and discusses some additional analyses conducted to test the robustness of these main findings. The final section concludes, discusses the limitations of our study and provides a (related) direction for future research.

## 2. Methods

### 2.1. Data

To test our research hypotheses, we analysed data from the European Social Survey, i.e. a cross-national survey that has been conducted since 2001. It measures the beliefs, preferences and behaviour of various populations in Europe. Every two years, fieldworkers survey randomly selected participants aged 15 and over by means of face-to-face interviews. For countries with fewer than two million residents, at least 800 individuals are interviewed; for countries with more than two million residents, the minimum number of respondents is 1500. For the present study, we used data from Round 6 (ESS6), data edition 2.2. These data were gathered in 2012 and 2013 in 29 countries: the (former) European Union (EU) countries—alphabetically ordered by their ISO 3166–1 alpha-3 code—Belgium, Bulgaria, Cyprus, Czech Republic, Germany, Denmark, Spain, Estonia, Finland, France, United Kingdom, Hungary, Ireland, Italy, Lithuania, Netherlands, Poland, Portugal, Slovakia, Slovenia and Sweden; and the non-European Union countries Albania, Iceland, Israel, Kosovo, Norway, Switzerland, Russian Federation and Ukraine. The ESS6 data have, in view of our research goals, the advantage that they contain information on individual volunteering as well as on income and health. We refer to Norwegian Centre for Research Data [32] for more general information on ESS6.

Column (2) of Table 1 defines the variables that we selected for the respondents in ESS6. Our main outcome variable, i.e. self-rated health, was measured based on the responses of the ESS6 respondents to the following question: “How is your health in general?” The participants could choose from five categories ranging from “very bad” to “very good”. This variable is the only proxy for general health available in ESS6.

**Table 1:** Summary statistics: Description of variables and summary statistics.

Variable	Definition	All		Non-volunteers		Volunteers		Difference: (7)–(5)
		Mean	SD	Mean	SD	Mean	SD	
(1)	(2)	(3)	(4)	(5)	(6)	(7)	(8)	(9)
Male gender	Whether respondent is male	0.461	0.498	0.458	0.498	0.472	0.499	0.015*** [2.591]
Age	Age of respondent	49.180	18.113	49.484	18.400	48.226	17.143	-1.257*** [6.156]
Education level								
Low education	Educational attainment of respondent: lower-secondary education or lower	0.256	0.436	0.276	0.447	0.194	0.396	-0.082*** [16.562]
Middle education	Educational attainment of respondent: upper-secondary education	0.381	0.486	0.393	0.488	0.344	0.475	-0.050*** [9.049]
High education	Educational attainment of respondent: post-secondary education or higher	0.363	0.481	0.331	0.471	0.462	0.499	0.131*** [24.178]
Migrant	Whether respondent’s mother was born in a foreign country	0.130	0.336	0.132	0.338	0.124	0.329	-0.008** [2.180]
Religiosity	Index for respondent’s religiosity going from 0 (not religious at all) to 10 (very religious)	4.770	3.077	4.665	3.072	5.101	3.067	0.436*** [12.585]
Volunteering	Whether respondent volunteers at least once every six months	0.241	0.428	0.000	0.000	1.000	0.000	-
Income	Index for respondent’s household net income going from 1 (lowest decile of the country) to 10 (highest decile of the country)	5.071	2.815	4.912	2.798	5.568	2.809	0.656*** [20.748]
Health	Index for respondent’s subjective general health going from 1 (very bad) to 5 (very good)	3.734	0.940	3.673	0.958	3.927	0.854	0.254*** [24.074]
Observations		42926		32568		10358		

Note: Test statistics are calculated to test whether the differences in column (9) are significantly different from 0. More concretely, for the continuous variables (age, religiosity, income and health) t-tests are calculated and for the binary variables (male gender, low education, middle education, high education, migrant and volunteering) two-proportion z-tests are calculated. These test statistics are between brackets.

*** indicates significance at the 1% level.

** indicates significance at the 5% level.

Self-rated measures are often criticised with respect to their validity, as respondents may lie or answer in a socially desirable way. However, following the World Health Organisation (WHO) definition of health in terms of “physical, mental and social well-being, and not merely the absence of disease and infirmity”, objectively measuring health is a difficult—nearly impossible—exercise. For this reason, and reasons of simplicity and cost, the subjective operationalisation of health has been commonly used in the literature [33–34]. More importantly, many studies have shown that self-rated health is not only a commonly used but also a valid predictor of actual health in general and mortality in particular [35–36]. In addition, measuring health from the perspective of the respondent yields an important advantage. It captures health indicators that are hard to measure by physical measurement, such as pain, suffering or depression [33]. Therefore, using self-rated health as an outcome variable is in line with the shift from problem-oriented to patient- and goal-oriented care as reported in many recent studies in health sciences [37–38].

For our income variable we used, by analogy with Day and Devlin [20], Day and Devlin [21] and Hackl et al. [23], the respondents’ net household income. This variable was measured in ESS6 as follows. The respondents were shown a card with the current (weekly, monthly and annual) income deciles in their country, ranging from the lowest to the highest decile. Each decile on this card was labelled by a letter. Then the respondents had to answer the following question: “Using this card, please tell me which letter describes your household’s total income, after tax and compulsory deductions, from all sources? If you don’t know the exact figure, please give an estimate. Use the part of the card that you know best: weekly, monthly or annual income.” This variable is the only proxy of income and earnings available in ESS6. It has the disadvantage that it is also affected by the earnings (and, *ipso facto*, potentially, the volunteering activities) of the respondents’ spouses. Still, this variable is an important proxy of the respondents’ labour market status and productivity: higher earnings of the respondent translate into a higher household income, *ceteris paribus*. Moreover, with respect to health-related investments and consumption, net household income is a more relevant determinant than individual earnings.

As about 11% of the respondents in ESS6 refused to answer the income question and an additional 8% of the respondents answered that they did not know their household’s net income, this variable was missing for a substantial number of individuals observed in ESS6. As a consequence, these individuals had to be excluded from our sample. We elaborate on the potential selectivity bias this may have yielded in Section 3.2.

Although they are of an ordinal nature in ESS6, throughout our analyses, we treat the health and income variables as continuous with the health variable ranging from 1 (very bad) to 5 (very good) and the income variable ranging from 1 (lowest decile) to 10 (highest decile). Treating ordinal variables as continuous is common and appropriate when ordinal variables have at least five categories [39–40].

With respect to our main explanatory variable, i.e. volunteering, we relied on the respondents’ answers to the question: “In the past 12 months, how often did you get involved in work for voluntary or charitable organisations?” The participants could choose from six categories ranging from “never” to “at least once a week”. In line with Baert and Vujić [41], we defined a volunteer as someone who was involved in volunteer activities at least once every six months. We discuss the sensitivity of our results with respect to this definition in Section 3.2.

In view of the potential endogeneity of volunteering with respect to income and self-rated health, in our models outlined in Section 2.3, we included the following exogenous variables as controls: gender, age, education level, migrant status and religiosity. With respect to educational attainment, we opted to control for the following three levels as observed in ESS6: “lower-secondary or lower”, “upper-secondary” and “post-secondary or higher”. In what follows, we will refer to these three levels as “low education”, “middle education” and “high education”. Migrant status was defined following Rumbaut [42]. More concretely, it was determined by the birthplace of the respondents’ mother. When the mother was born in the host country, the participant was considered “native”; otherwise she/he was considered to be a migrant. Finally, we included an index of individual (self-rated) religiosity, i.e. the ESS6 participants’ answer to the following question: “Regardless of whether you belong to a particular religion, how religious would you say you are?” The participants had to score their religiosity on an index ranging from 0 (not religious at all) to 10 (very religious).

We elaborate on analyses with an extended set of control variables in Section 3.2. In addition, in Section 4 we discuss how other potential determinants of volunteering, income and health, for which we were not able to control, stand in the way of the causal interpretation of our findings. For this reason, throughout this article, we refer to our results as associations and not as (causal) effects.

### 2.2. Descriptive statistics

Table 1 reports descriptive statistics for the aforementioned variables. We separately report statistics on the full sample of 42926 individuals in the ESS6 for whom all the variables mentioned in Section 2.1 were observed, on the subsample of non-volunteers and on the subsample of volunteers. In total, 24.1% of the subjects in our sample volunteered following the aforementioned definition and 75.9% did not volunteer. This fraction of volunteering individuals is heterogeneous across countries. Whereas in Germany, the Netherlands and Norway more than 40% of them volunteered, in Bulgaria, Hungary and Lithuania fewer than 10% engaged in volunteering activities at least once every six months.

The subsample of volunteers comprises relatively more male, highly educated and religious individuals and fewer individuals at an older age and migrants. As these variables might also affect income and health, controlling for them in the context of our study is relevant. In addition, and in line with our expectations, the descriptive statistics with respect to both income and health are somewhat more beneficial among the volunteers. However, this comparison does not take selection on the aforementioned sociodemographic background characteristics into account. Nor does it provide an insight into the association between income and health and, *ipso facto*, the income channel through which volunteering might affect health. The mediation analysis we apply in this research takes the aforementioned selection problem into account and incorporates the potential indirect association between volunteering and health via income.

### 2.3. Statistical analyses

To test H1, we regressed self-rated health on volunteering status. In this regression analysis, the exogenous variables mentioned in Section 2.1 were controlled for. In addition, given the heterogeneity of countries with respect to self-rated health and volunteering (see Section 2.2), country dummies were included.

**Fig 1:**
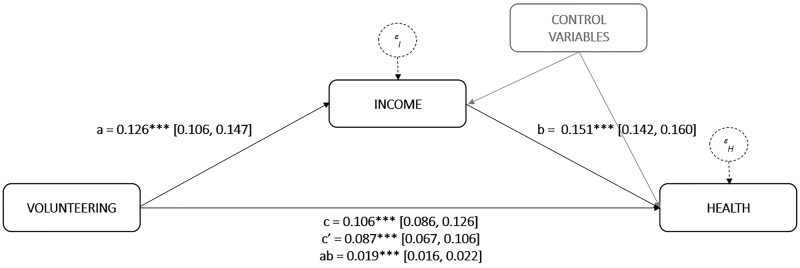
Mediation analysis in the form of a statistical diagram. Note: The presented statistics are coefficient estimates and 95% confidence intervals between brackets for a mediation model (with income as mediator between volunteering and health) using the PROCESS procedure as described in Hayes [43]. The confidence interval for the mediation outcome (ab) is based on 10000 bootstrap samples. *** indicates significance at the 1% level. See Table 1 for the definitions of the variables included. The variables religiosity, income and health are standardised.

To test H2, we performed a mediation analysis following the state-of-the-art PROCESS procedure as described in Hayes [43]. In our mediation model, which is schematised in Fig 1, volunteering is associated with health in both a direct way and an indirect way via income. In addition, both income and health are explained by the exogenous variables and country dummies adopted in the aforementioned regression analysis. The PROCESS procedure allowed us to split the total association between volunteering and health, as estimated in the regression analysis, in a direct association and an indirect association via income.

In both analyses, the volunteering variable was included, in line with our definition mentioned in Section 2.1, as a binary variable (equal to 1 in case the respondent was engaged in volunteering and 0 otherwise) and the health and income variables as continuous variables. However, in Section 3.2 we discuss analyses in which other specifications were adopted. To be able to convey the practical significance of the measured associations, all continuous indices (i.e. health, income and religiosity) were standardised (by subtracting their sample mean and dividing the result by these variables’ sample standard deviation) in preparation of the analyses [44–45].

Both statistical models were estimated in SPSS (IBM, version 24.0.0.0). The confidence interval for the mediation measure was based on 10000 bootstrap samples.

## 3. Results

### 3.1. Main results

Table 2 presents the estimation results for our regression analysis. We find, in line with H1, a positive association between volunteering and health. Volunteers have a self-rated health which is on average 0.106 standard deviations higher than those who do not volunteer, *ceteris paribus*. In other words, health is about a ninth of a standard deviation higher for volunteers. This association is comparable in size to the health gains of being a man (associated with a 0.086 standard deviation increase in self-rated health; compared to being a woman), being five years younger (associated with a 0.105 standard deviation increase in self-rated health; 0.105 = -5 x -0.021) or being a native (associated with a 0.111 standard deviation increase in self-rated health; compared to being a migrant). Moreover, this direct association between volunteering and health is statistically significantly different from 0 at the 1% significance level (p = 0.000).

**Table 2:** Estimation results: Regression analysis.

Explanatory variables	Outcome variable: Health
Male gender	0.086*** [0.069, 0.102]
Age	-0.021*** [-0.022, -0.021]
Education: low education	-0.353*** [-0.375, -0.330]
Education: middle education	-0.163*** [-0.182, -0.143]
*Education*: *high education (reference)*	
Migrant	-0.111*** [-0.137, -0.085]
Religiosity	0.003 [-0.006, 0.011]
Volunteering	0.106*** [0.086, 0.126]
Constant	1.065*** [1.009, 1.122]
Country fixed effects	Yes
R²	0.276
Observations	42926

Note: The presented statistics are coefficient estimates and 95% confidence intervals between brackets for a linear regression model controlling for country fixed effects.

*** indicates significance at the 1% level. See Table 1 for the definitions of the variables included. The variables religiosity and health are standardised.

The results of our mediation analysis are depicted in Fig 1. The full estimation results are shown in Table 3. The total association between volunteering and health (c = 0.106; p = 0.000) is very similar to the related coefficient in our regression analysis. This total association can be decomposed in a direct association and an indirect association via income. The direct association, indicating whether volunteering is associated with health after controlling for income as a mediator, is substantial (c’ = 0.081; p = 0.000).

**Table 3:** Estimation results: Mediation analysis.

Explanatory variables	Outcome variables	
	Income	Health
Male gender	0.163*** [0.146, 0.180]	0.061*** [0.045, 0.077]
Age	-0.009*** [-0.010, -0.009]	-0.020*** [-0.021, -0.020]
Education: low education	-0.785*** [-0.808, -0.762]	-0.234*** [-0.258, -0.211]
Education: middle education	-0.473*** [-0.493, -0.453]	-0.092*** [-0.111, -0.072]
*Education*: *high education (reference)*		
Migrant	-0.120*** [-0.147, -0.094]	-0.093*** [-0.118, -0.067]
Religiosity	-0.025*** [-0.034, -0.016]	0.006 [-0.026, 0.015]
Volunteering	0.126*** [0.106, 0.147]	0.087*** [0.067, 0.106]
Income	-	0.151*** [0.142, 0.160]
Constant	0.116*** [0.058, 0.174]	1.048*** [0.992, 1.104]
Country fixed effects	Yes	Yes
R²	0.486	0.541
Observations	42926	

Note: The presented statistics are coefficient estimates and 95% confidence intervals between brackets for a mediation model (with income as mediator between volunteering and health) using the PROCESS procedure as described in Hayes [43]. The confidence interval for the mediation effect is based on 10000 bootstrap samples.

*** indicates significance at the 1% level. See Table 1 for the definitions of the variables included. The variables religiosity, income and health are standardised.

Concerning the association between volunteering and income, we find, in line with the surplus of volunteering found in the economics literature, a significantly positive association. Income is 0.126 standard deviations higher among volunteers than among those who do not volunteer (a = 0.126; p = 0.000). In addition, consistent with the well-established positive association between income and health, we find that health is higher when income is higher. More concretely, health increases by 0.151 standard deviations for every one standard deviation increase in income (b = 0.151; p = 0.000). Multiplying the latter two estimation coefficients yields a significantly positive mediation outcome (ab = 0.019; p = 0.000). So, volunteering is indirectly associated with a 0.019 standard deviation increase in self-rated health via income. As a consequence, notwithstanding its low practical significance, this result confirms H2. The association between volunteering and health is partially mediated by household income.

Taken together, our estimation results show that the direct association between volunteering and self-rated health is more substantial than their indirect association via household income. The mediation ratio (i.e. the ratio of the indirect association to the total association; [45]) is 0.179 (i.e. 0.019/0.106). So, income mediates 17.9% of the total association between volunteering and self-rated health (while the direct association accounts for more than four fifths).

### 3.2. Sensitivity analyses

In this subsection, we report on some alternative analyses we conducted to test the robustness of our main results discussed in the former subsection.

Firstly, as mentioned in Section 2.1, our income variable was missing for a substantial number of individuals observed in ESS6. More concretely, among the respondents for whom no other variables included in our model were missing—these other variables were missing in less than 1% of cases—the income variable was registered for 42926 respondents and missing for 10051 respondents. This implies that our research sample was potentially a selective subsample of the full sample of randomly selected individuals in ESS6. In Table 4 we compare the descriptive statistics of the respondents for which the income variable was observed with the corresponding statistics of those for which the income variable was not observed. This table shows that the individuals in the sample used for our main analysis were, compared to those who were excluded based on their missing income variable, more often (i) male, (ii) old(er), (iii) highly educated, (iv) native, (v) volunteering and (vi) in bad health.

**Table 4:** Test for sample selectivity.

Variable	Research sample (without individuals with missing observation for income)		Sample with missing observation for income		Difference: (2)–(4)
	Mean	SD	Mean	SD	
(1)	(2)	(3)	(4)	(5)	(6)
Male gender	0.461	0.498	0.432	0.495	0.029*** [5.228]
Age	49.180	18.113	44.697	20.074	4.484*** [21.871]
Education level					
Low education	0.256	0.436	0.327	0.469	-0.071*** [14.438]
Middle education	0.381	0.486	0.374	0.484	0.007 [1.343]
High education	0.363	0.481	0.299	0.458	0.063*** [12.009]
Migrant	0.130	0.336	0.137	0.344	-0.007** [1.992]
Religiosity	4.770	3.077	4.734	3.083	0.036 [1.046]
Volunteering	0.241	0.428	0.216	0.412	0.025*** [5.268]
Health	3.734	0.940	3.848	0.923	-0.113*** [10.916]
Observations	42926		10051		

Note: Test statistics are calculated to test whether the differences in column (9) are significantly different from 0. More concretely, for the continuous variables (age, religiosity and health) t-tests are calculated and for the binary variables (male gender, low education, middle education, high education, migrant and volunteering) two-proportion z-tests are calculated. These test statistics are between brackets.

*** indicates significance at the 1% level.

** indicates significance at the 5% level.

In a second robustness check, we restricted our sample to individuals in the labour force, i.e. those who indicated “employment” or “unemployment” (not in employment but searching for work) as their main activity during the last seven days. We did this as for other groups in ESS6 (those with “permanently sick or disabled”, “retired”, “community or military service”, “housework” or “other” as a main activity), their income is less dependent on their own choices (among which is volunteering). This alternative strategy yielded a more homogeneous research population at the cost of introducing a selection problem.

Thirdly, we re-estimated our model using alternative variables capturing volunteering status (e.g., a continuous variable based on the original categorical variable available in ESS6, and binary variables indicating individuals involved in volunteer activities at least once every week, every month or every three months instead of every six months, i.e. the threshold used for our benchmark definition).

Fourthly, the following additional variables were included as sociodemographic controls: maternal and paternal education level, an indicator for having a partner, number of household members and a dummy for being a second generation migrant. These variables were relevant to control because they are potential determinants of volunteering, health and—especially—household income, but we did not include them in the main analyses as they turned out to be missing for many observations or could not be considered as exogenous with respect to our three outcome variables.

However, none of the latter three analyses, the results of which are available upon request, led to conclusions other than those discussed in Section 3.1.

## 4. Conclusion

In this article, we studied the association between volunteering and self-rated health. More concretely, we contributed to the literature by investigating both the direct association between volunteering and health and their indirect association via (household) income. To this end, we proposed a state-of-the-art mediation model. This model was estimated based on data from the sixth round of the European Social Survey (ESS6), gathered in 2012 and 2013 within 29 European countries. From these data, observations for all 42926 individuals who revealed their household income were used.

We found that, overall, volunteers have a health score which is statistically significantly higher than those who do not volunteer. This total association turned out to be substantial: it corresponded in size to, e.g., the health gains of a five years younger age. When decomposing this total association between volunteering and health in a direct association and an indirect association via income, we found that the former accounts for more than four fifths of the total association while the latter accounts for less than one fifth.

In our Data subsection, we acknowledged limitations of our study related to (i) the use of a self-rated scale of health and (ii) the selectivity of our sample due to the missing observations in ESS6 with respect to household income. We end this article by discussing an additional important research limitation. The choice to volunteer is endogenous with respect to (labour market and) health-related outcomes. Individuals with particular characteristics select themselves into volunteer work and these characteristics might also be correlated with (income and) health. Without adequately controlling for this selection problem, research results cannot be given a causal interpretation. Therefore, we controlled for various sets of important confounders of the relationship under investigation, ran analyses on different samples, compared different definitions and proxies for our variables and used different specifications of our models. Still, it is likely that preferences and behaviour related to volunteering for which we were not able to control may affect (income and) health outcomes and, *ipso facto*, stand in the way of a causal interpretation of our findings. Given the deep rootedness of this endogeneity problem, we believe that only field experiments in which volunteering activities—or eligibility for these activities—are randomly assigned to individuals guarantee perfect causal inference. As a consequence, we look forward to trials in the sense of Tan et al. [11] and Carlson et al. [13] that innovate by measuring not only the total causal impact of particular prosocial engagements on health, but also decomposing this impact into a direct effect and an indirect effect via income (or other economic outcomes).
